# Supplementary material for: Causal Relationship between Aging and Anorexia Nervosa: A White-Matter-Microstructure-Mediated Mendelian Randomization Analysis
Source: Biomedicines. 2024 Aug 16;12(8):1874. doi: 10.3390/biomedicines12081874 (PMC11351342; doi:10.3390/biomedicines12081874)
Supplement: Supplementary file 1 [file biomedicines-12-01874-s001.zip › Supplementary Figure/Figure S4/Singe SNP plot of MR analysis for TL to ubm-b-1830.pdf]

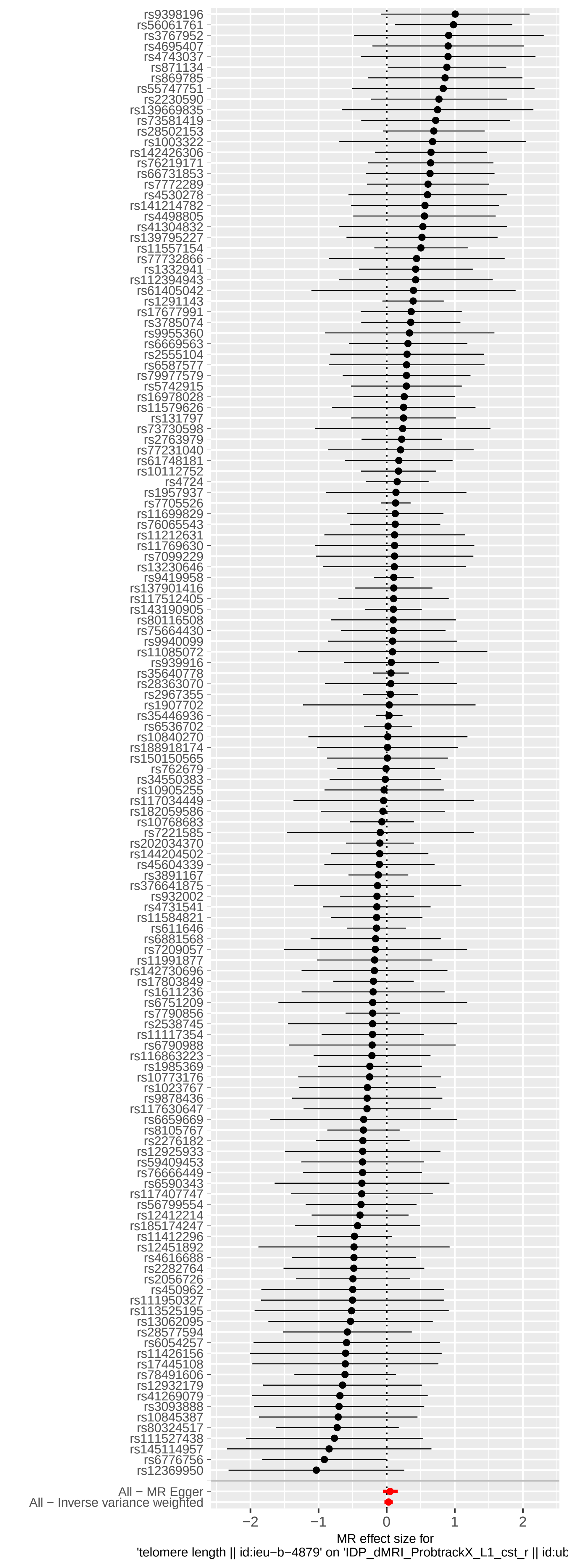
rs9398196
rs56061761
rs3767952
rs4695407
rs4743037
rs871134
rs869785
rs55747751
rs2230590
rs139669835
rs73581419
rs28502153
rs1003322
rs142426306
rs76219171
rs66731853
rs7772289
rs4530278
rs141214782
rs4498805
rs41304832
rs139795227
rs11557154
rs77732866
rs1332941
rs112394943
rs61405042
rs1291143
rs17677991
rs3785074
rs9955360
rs6669563
rs2555104
rs6587577
rs79977579
rs5742915
rs16978028
rs11579626
rs131797
rs73730598
rs2763979
rs77231040
rs61748181
rs10112752
rs4724
rs1957937
rs7705526
rs11699829
rs76065543
rs11212631
rs11769630
rs7099229
rs13230646
rs9419958
rs137901416
rs117512405
rs143190905
rs80116508
rs75664430
rs9940099
rs11085072
rs939916
rs35640778
rs28363070
rs2967355
rs1907702
rs35446936
rs6536702
rs10840270
rs188918174
rs150150565
rs762679
rs34550383
rs10905255
rs117034449
rs182059586
rs10768683
rs7221585
rs202034370
rs144204502
rs45604339
rs3891167
rs376641875
rs932002
rs4731541
rs11584821
rs611646
rs6881568
rs7209057
rs11991877
rs142730696
rs17803849
rs1611236
rs6751209
rs7790856
rs2538745
rs11117354
rs6790988
rs116863223
rs1985369
rs10773176
rs1023767
rs9878436
rs117630647
rs6659669
rs8105767
rs2276182
rs12925933
rs59409453
rs76666449
rs6590343
rs117407747
rs56799554
rs12412214
rs185174247
rs11412296
rs12451892
rs4616688
rs2282764
rs2056726
rs450962
rs111950327
rs113525195
rs13062095
rs28577594
rs6054257
rs11426156
rs17445108
rs78491606
rs12932179
rs41269079
rs3093888
rs10845387
rs80324517
rs111527438
rs145114957
rs6776756
rs12369950
All − MR Egger
All − Inverse variance weighted
−2
−1
0
1
2
MR effect size for
'telomere length || id:ieu−b−4879' on 'IDP_dMRI_ProbtrackX_L1_cst_r || id:ub
